# Depletion of HDAC6 Enhances Cisplatin-Induced DNA Damage and Apoptosis in Non-Small Cell Lung Cancer Cells

**DOI:** 10.1371/journal.pone.0044265

**Published:** 2012-09-05

**Authors:** Lei Wang, Shengyan Xiang, Kendra A. Williams, Huiqin Dong, Wenlong Bai, Santo V. Nicosia, Saadi Khochbin, Gerold Bepler, Xiaohong Zhang

**Affiliations:** 1 Department of Pathology and Cell Biology, University of South Florida, Morsani College of Medicine, Tampa, Florida, United States of America; 2 Program of Molecular Oncology, H. Lee Moffitt Cancer Center and Research Institute, Tampa, Florida, United States of America; 3 Experimental Therapeutics, H. Lee Moffitt Cancer Center and Research Institute, Tampa, Florida, United States of America; 4 French National Institute of Health and Medical Research, The Albert Bonniot Institute, Grenoble, France; 5 Department of Oncology, Karmanos Cancer Institute, Detroit, Michigan, United States of America; Technische Universität München, Germany

## Abstract

Histone deacetylase inhibitors (HDACi) are promising therapeutic agents which are currently used in combination with chemotherapeutic agents in clinical trials for cancer treatment including non-small cell lung cancer (NSCLC). However, the mechanisms underlying their anti-tumor activities remain elusive. Previous studies showed that inhibition of HDAC6 induces DNA damage and sensitizes transformed cells to anti-tumor agents such as etoposide and doxorubicin. Here, we showed that depletion of HDAC6 in two NSCLC cell lines, H292 and A549, sensitized cells to cisplatin, one of the first-line chemotherapeutic agents used to treat NSCLC. We suggested that depletion of HDAC6 increased cisplatin-induced cytotoxicity was due to the enhancement of apoptosis via activating ATR/Chk1 pathway. Furthermore, we showed that HDAC6 protein levels were positively correlated with cisplatin IC_50_ in 15 NSCLC cell lines. Lastly, depletion of HDAC6 in H292 xenografts rendered decreased tumor weight and volume and exhibited increased basal apoptosis compared with the controls in a xenograft mouse model. In summary, our findings suggest that HDAC6 is positively associated with cisplatin resistance in NSCLC and reveal HDAC6 as a potential novel therapeutic target for platinum refractory NSCLC.

## Introduction

Lung cancer remains the leading cause of cancer death for both men and women in the United States, claiming more lives annually than the next three causes of cancer death (cancers of the breast, colon and prostate) combined [1]. NSCLC accounts for more than 80% of all lung cancers. Survival rates for patients with NSCLC remain extremely low, with only 16% of patients alive 5 years after a lung cancer diagnosis. Although this poor prognosis is explained in part by the large numbers of patients who present with advanced disease, even patients identified at an early-stage experience high rates of relapse in spite of adequate surgical resection [2].

Several large randomized trials have demonstrated modest improvements in long-term survival with adjuvant cisplatin-based chemotherapy [3]–[6]. On the basis of these studies, adjuvant chemotherapy has become the standard of care for patients with stage II and III NSCLC. Given that a relatively small population appears to benefit from chemotherapy, many patients are subjected to toxic treatment without clinical benefit. A better understanding of the mechanisms of resistance to platinum-based chemotherapy is required and strategies are needed to identify patients unlikely to benefit from treatment. Novel methods of overcoming platinum resistance may be targeted to these populations.

HDACs, a class of enzymes that remove acetyl groups from ε-N-acetyl lysine amino acid on histones or other non-histone proteins, play important roles in cell growth, apoptosis, DNA damage, etc. The mammalian HDACs are divided into four classes: class I (HDACs 1, 2, 3, and 8), class II (HDACs 4, 5, 6, 7, 9, and 10), class III (SIRTs 1, 2, 3, 4, 5, 6, and 7), and class IV (HDAC11) [7], [8]. Class I HDACs localize mainly in the nucleus and are found in repressive complexes such as Sin3, NuRD, CoREST, PRC2, N-CoR, and SMRT complexes, which deacetylate histones and other nuclear proteins. Class II HDACs are further divided into IIa and IIb subclasses, and these members exhibit tissue-specific expression. Class III members are Sir2-related NAD^+^-dependent deacetylases. HDAC11 is the only member of the Class IV family due to its low sequence similarity to class I and class II members.

HDAC6 belongs to the class IIb HDACs. It was cloned as a mammalian homolog of yeast HDA1 from mouse and human, respectively [9], [10]. Uniquely, HDAC6 contains two functional tandem deacetylase domains, termed DAC1 and DAC2, or DD1 and DD2, as well as a ZnF-UBP domain which is a zinc finger containing region that is homologous with the non-catalytic domain of several ubiquitin-specific proteases (USPs) [11]. HDAC6 ZnF-UBP domain is able to bind mono-, or poly-ubiquitin as well as ubiquitinated proteins [11]–[13]. Substrates of HDAC6 include cytosolic proteins such as α-tubulin, hsp90, cortactin, etc [14]–[16]. HDAC6 also acts in ubiquitin-dependent autophagy by allowing the processing or degradation of protein aggregates [17]. Additionally, HDAC6 is involved in misfolded protein induced cell stress [18]. HDAC6 is now considered as a master regulator of cell response to cytotoxic assaults [19]. A recent report has shown that HDAC6 is involved in DNA-damaging agents induced genotoxic stress [20]. However, the underlying mechanisms are far from clear.

HDACs expressions are altered in numerous cancers. For example, overexpression of HDAC1, HDAC2, HDAC3, and HDAC6 has been observed in colon, breast, prostate, cervical and gastric cancers [21]–[28]. Inhibiting HDAC enzymes by HDAC inhibitors has emerged as a promising approach for the treatment of cancers [29]–[31]. HDAC inhibitors alter the acetylation status of chromatin and other non-histone proteins, resulting in changes in gene expression, induction of apoptosis, growth arrest, and cell terminal differentiation [31]. In particular, HDAC inhibitors are promising adjuvant drugs used in combination with platinum-based chemotherapy in several cancers including lung cancer [32].

In this study, we have shown that HDAC6 confers cisplatin resistance in NSCLC cell lines. Knockdown of HDAC6 or inhibition of HDAC6 activity by HDAC6-specific inhibitor tubastatin A in NSCLC cell lines A549 and H292 sensitized the cells to cisplatin treatment. In addition, a positive and significant correlation between HDAC6 protein level and IC_50_ of cisplatin was found in 15 NSCLC cell lines. Moreover, H292 xenografts with HDAC6 depletion displayed smaller tumor weight and volume, as well as increased basal apoptosis compared with control xenografts. Altogether, our results suggest that development of clinical relevant HDAC6-selective inhibitors will be beneficial to be used in combination with platinum-based therapy in NSCLC.

## Materials and Methods

### Chemicals and Reagents

A 10 mM stock solution of vorinostat (Selleck chemicals) was prepared in dimethyl sulfoxide (DMSO) and diluted as the needed concentrations in cell culture medium. Tubastatin A was purchased from BioVision. A 15 mM stock solution of tubastatin A was prepared in DMSO. Cisplatin and paclitaxel were purchased from Sigma. Cisplatin was prepared as a 50 mM stock solution in dimethylformamide (DMF) and paclitaxel as a 10 mM stock solution in DMSO. Antibodies against phosphorylation of histone H2AX (γH2AX) (20E3), phosphorylated ATR (Ser428) (#2853), phosphorylated Chk1 (Ser296) (#2349), phosphorylated p53 (Ser15) (16G8), Anti-ATR (#2790), anti-caspase 3 (8G10), PARP-1 (#9524S), and α-tubulin (1H10) were purchased from Cell Signaling Technology, while anti-acetylated tubulin and anti-β-actin were from Sigma. Anti-Chk1 (2G1D5), anti-p53 (DO-1) and anti-HDAC6 (H-300) antibodies were purchased from Santa Cruz Biotechnology.

### Cell Lines and the Generation of Scramble and HDAC6 Knockdown H292 Cell Lines

NSCLC cell lines ADLC, A549, EPLC, H23, H292, H358, H441, H460, H522, H661, H820, H1650, H1975, H2122, and H2172 were obtained from the American Type Culture Collection (ATCC). Cells were cultured in RPMI 1640 medium with penicillin (100 U/ml), streptomycin (100 µg/ml) and 10% fetal bovine serum (FBS) and incubated at 37°C with 5% CO_2_. A549 HDAC6 knockdown (A549-HD6KD) and control (A549-Sup) stable cell lines were kindly provided by Dr. Tso-Pang Yao as described by Kawaguchi et al. [18]. H292 scramble and HDAC6 knockdown stable cell lines were clonally selected by 0.5 µg/ml puromycin. First, H292 cells were transiently transfected with control vector pRS (Cat.# TR20003) or shRNA vector against HDAC6 (recognize sequence 5-AGGTCTACTGTGGTCGTTACATCAATGGC-3′, tube ID:TI349960, ORIGENE). Twenty-four hours after transfection, cells were split to duplicate plates of 1∶20 in RPMI1640 medium containing 0.5 µg/ml puromycin. Puromycin was replenished every 2 days to maintain sufficient level of selection pressure. The well-isolated single clones were transferred into 24-well plates. The knockdown effect was verified by Western Blotting analysis using anti-HDAC6 antibodies.

### MTT Assays

Cell growth and viability were evaluated by MTT assay. Briefly, cells were seeded in sextuplet into 96-well flat bottom plates at a density of 5×10^3^ cells/well. The drugs were added at the indicated concentrations 24 h after seeding while vehicle was added as control. At the indicated days, cells were incubated with 3-(4, 5-dimethylthiszol-x-yl)-2, 5-diphenytetrazolium bromide (MTT) (Sigma) solution for 4 h, then supplemented with 100 µl of DMSO and shaken for 15 min. The absorbance of cisplatin-exposed cultures was measured using a multi-well spectrophotometer at 550 nm with a 630 nm reference. The results were presented as percent absorbance relative to vehicle control cultures.

### Cell Cycle Analysis

H292 cells were harvested and gently resuspended into single cell suspension in Fluorescence Activated Cell Sorting (FACS) buffer (PBS containing 2% FBS). The cells were washed with PBS twice and fixed in cold 70% ethanol overnight. Cells were then washed twice with cold PBS again, resuspended in 50 µl PBS plus 3.3 µl of RNase A solution (30 µg/ml) and incubated for 30 min at 4°C. The suspension was added with 450 µl FACS buffer and 25 µl propidium iodide (PI, 1 mg/ml) followed by incubation at 4°C for 30 minutes. Cells were then analyzed with FACS machine immediately. Results were analyzed with FlowJo v8.3.3 software.

### Immunoblotting Analysis

Whole-cell extracts were prepared by adding RIPA buffer (25 mM Tris-HCl pH 7.6, 150 mM NaCl, 1% NP-40, 1% sodium deoxycholate, 0.1% SDS, and protease inhibitor cocktail). Protein concentration of all samples was determined by Bradford reagent (Bio-Rad). Proteins were resolved on 8% or 12% SDS-PAGE gels and transferred to nitrocellulose membranes. The membranes were probed with primary antibodies and horseradish peroxidase (HRP) conjugated secondary antibodies (GE healthcare). Lastly, the blots were visualized using Chemiluminescent Detection Kit (Pierce).

### Comet Assays

Following cisplatin treatment, H292-pRS control or HD6-KD cells were irradiated with 10 Gy of gamma radiation to introduce random single-strand breaks. Basically, the more crosslinks introduced by cisplatin, the fewer single-strand breaks will be detected in irradiated cells. The comet assays were performed using an OxiSelect™ Comet Assay kit (Cell Biolabs, Inc.) according to the manufacturer’s protocol.

### Immunofluoresence Microscopy

Cells cultured on chamber slides (Chamber Slide System Lab-TekII) were washed with PBS and fixed in 4% paraformaldehyde for 10 min at room temperature. Fixation was stopped with PBS containing 1% glycine. Cells were permeabilized using 1% glycine/0.5% Triton X-100 solution at room temperature for 15 min. After blocking with 5% bovine serum albumin (BSA) for 1 hr, cells were incubated in PBS containing 0.2% Triton X-100, 1% BSA, and the anti-γH2AX antibody (20E3) overnight at 4°C. Cells were then washed with PBST (PBS containing 0.1% Tween) followed by incubation with secondary antibody (in PBS containing 1% BSA) for 45 min. Finally, cells were washed with PBS containing 0.1% Tween and then PBS alone. The slides were dried and mounted with Vectashield® mounting medium containing DAPI (Vector Laboratories, Burlingame, CA).

### Colony Formation Assay

Briefly, cells were seeded in triplicates (1,000 cells per 60 mm tissue culture dish) and incubated overnight at 37°C allowing cells to attach to the dishes. Cells were then treated with vehicle control or cisplatin for 8 days. Afterwards, cells were washed with PBS, fixed with 4% paraformaldehyde for 15 min, and stained with crystal violet (0.5% crystal violet, 1% paraformaldehyde and 20% methanol in PBS) for 30 min. Colonies on each plate were counted and cell survival after cisplatin treatment was expressed as a percentage of the number of surviving colonies on the control plates.

### Tumor Growth

All procedures with mice were performed under a protocol titled: Role of HDAC6 in Platinum Resistance of Non-Small Cell Lung Cancer which was approved by an Institutional Animal Care and Use Committee (IACUC) in the Division of Research Integrity and Compliance at the University of South Florida on 10/19/2011 (Protocol #R4064). The 5- to 6-week-old female athymic nude mice (Nu/Nu mice) weighing ∼20 g were purchased from the National Cancer Institute (NCI). H292-pRS control cells (5×10^6^ cells/100 µl in RPMI 1640 medium plus 50% matrigel) were injected subcutaneously into the left flank and the same amount of H292-HD6KD cells were injected into the right flank. Tumors were allowed to grow for six weeks. Tumor sizes were recorded every week. Tumor volume was calculated using the formula V = (L×W^2^)×0.5 (V = volume, L = length, W = width) [33]. Mean tumor weight was also calculated after harvesting tumors.

### Immunohistochemical Staining

H292 control and HDAC6 knockdown xenografts were harvested and fixed in formalin, then embedded in paraffin and sectioned. Tissue microarray of 12 xenografts of H292-pRS and H292-HD6KD pairs were prepared by Moffitt histology core facility using Beecher Instrument Tissue Arrayer. Slides were stained using a Ventana Discovery XT automated system (Ventana Medical Systems, Tucson, AZ) as per manufacturer's protocol with proprietary reagents. Briefly, slides were deparaffinized on the automated system with EZ Prep solution (Ventana). Heat-induced antigen retrieval method was used in Cell Conditioning 1 (Ventana). The rabbit primary antibody that reacts to cleaved caspase 3, (#9661, Cell Signaling, Danvers, MA) or Ki67 (#M3060, Spring Bioscience) was used at a 1∶400 or 1∶100 concentration in Dako antibody diluent (Carpenteria, CA) and incubated for 60 min. The Ventana anti-rabbit secondary antibody was used for 16 min. The detection system used was the Ventana OmniMap kit, and slides were then counterstained with Hematoxylin. Slides were then dehydrated and coverslipped as per normal laboratory protocol.

A Tissue MicroArray (TMA) slide stained against cleaved caspase 3 has been digitally scanned using the Aperio™ (Vista, CA) ScanScope XT with a 200x/0.75NA objective lens at a rate of 4 minutes per slide via Basler tri-linear-array. Image analysis was performed using an optimized Aperio PositivePixelCount ® v9.0 algorithm with the following optimized thresholds [HueValue = 0.1; HueWidth = 0.5; Iwp(High) = 220; Iwp(Low)/Ip (High) = 175; Ip(Low)/Isp (High) = 100; Isp(Low) = 0; Inp(High) = −1] to segment positive pixels of various intensities. The percent of positivity has been quantified by the number of cells exhibiting positive stain as a percentage of total tumor cell count. The staining intensity was thresholded as parametertized above into negative (0), low (1+), moderate (2+) and strong positive (3+) by mean stain density (0–255 dynamic range) for each TMA core. The training algorithm developed was quality controlled by a practicing pathologist.

### Statistical Analysis

All assays were performed in at least three independent experiments. The data were presented as mean ± SEM, and statistical comparisons between groups were performed using one-way ANOVA followed by Dunnet test. A *p*-value <0.05 was considered statistically significant. Spearman coefficient and *p*-value for the correlation between HDAC6 level and cisplatin IC_50_ were calculated by SAS software.

## Results

### HDAC6 is Associated with Cisplatin-induced Cytotoxicity in NSCLC Cell Lines

**Figure 1:**
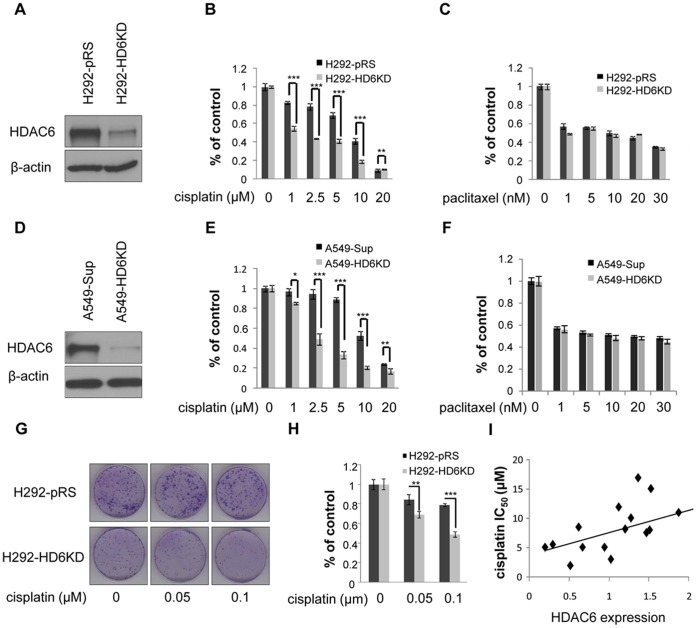
HDAC6 level is associated with cisplatin sensitivity in NSCLC cell lines. A , H292 cells were transfected with scramble shRNA or shRNA against HDAC6 to generate H292-pRS or H292-HD6KD stable clone, respectively. HDAC6 expression in control (H292-pRS) or HDAC6 knockdown (H292-HD6KD) cells was detected by anti-HDAC6 Western blotting analysis (upper panel). Anti-β-actin Western blotting was performed to ensure equal loading of protein (lower panel). **B**, Knockdown of HDAC6 sensitized H292 cells to cisplatin treatment. MTT assays were performed using H292-pRS or H292-HD6KD cells exposed to vehicle control (0 µM cisplatin) or indicated concentrations of cisplatin for 3 days. **C**, Knockdown of HDAC6 did not sensitize H292 cells to paclitaxel treatment. MTT assays were performed using H292-pRS or H292-HD6KD cells treated with vehicle control (0 nM paclitaxel) or indicated concentrations of paclitaxel for 3 days. **D**, HDAC6 was efficiently depleted in A549 cells. HDAC6 expression in control (A549-Sup) or HDAC6 knockdown cells (A549-HD6KD) was detected by anti-HDAC6 Western blotting analysis (upper panel). The anti-β-actin Western blotting analysis was also performed to ensure equal loading (lower panel). **E**, Knockdown of HDAC6 sensitized A549 cells to cisplatin treatment. MTT assays were performed using A549-Sup or A549-HD6KD cells treated with vehicle control or indicated concentrations of cisplatin for 3 days. **F**, Knockdown of HDAC6 did not sensitize A549 cells to paclitaxel treatment. MTT assays were performed using A549-Sup or A549-HD6KD cells treated with vehicle control or indicated concentrations of paclitaxel for 3 days. **G**, Colony formation assays were carried out with H292-pRS and H292-HD6KD cells for 8 days with vehicle control (0 µM cisplatin) or indicated concentrations of cisplatin. **H**, The quantitative results were obtained by calculating colonies from **G**. Columns, mean from three or six duplicates; bars, SEM (*, P<0.05; **, P<0.01; ***, P<0.001). **I**, HDAC6 levels positively correlate with cisplatin IC_50_ in a panel of NSCLC cell lines. HDAC6 protein levels were obtained by Western blotting analyses of 15 NSCLC lines (ADLC, A549, EPLC, H23, H292, H358, H441, H460, H522, H661, H820, H1650, H1975, H2122, and H2172) and quantified by Quantity One software. Cisplatin IC_50_ was calculated from MTT assays after treatment of these cells for 3 days. The correlation between HDAC6 protein level and cisplatin IC_50_ was analyzed by SAS software. A dot plot was used to illustrate the positive correlation between HDAC6 level and cisplatin IC_50_. Spearman coefficient was 0.64875 and *p*-value was 0.0089.

To characterize the role of HDAC6 in cisplatin resistance, two pairs of HDAC6 knockdown cell lines from H292 and A549 were established (Figure 1A and 1D). Cytotoxicity of cisplatin in control and HDAC6 knockdown pairs (H292-pRS and H292-HD6KD; A549-Sup and A549-HD6KD) were measured by MTT assays. As shown in Figure 1B and 1E, A549 and H292 cells depleted of HDAC6 displayed enhanced cytotoxicity after cisplatin treatment for 3 days, compared with those of control cells. To determine the long-term effect of HDAC6 knockdown on cell survival, we performed colony formation assays. As shown in Figure 1G and 1H, depletion of HDAC6 significantly decreased the numbers of colony formation in H292 cells upon cisplatin treatment. Interestingly, knockdown of HDAC6 does not enhance paclitaxel-induced cytotoxicity in A549 and H292 cells, suggesting that the increased sensitivity to cisplatin by depletion of HDAC6 was drug specific (Figure 1C and 1F). To examine whether depletion of HDAC6 can re-sensitize cisplatin resistant cells, C13 cell line, a cisplatin resistant ovarian cancer cell line derived from OV2008 [34], was stably transfected with control or HDAC6 shRNA to establish control and HDAC6 knockdown cell lines. As shown in Figure S1, depletion of HDAC6 in C13 cells dramatically reduced the cisplatin IC_50_ compared with control cells. In order to extend our findings, we measured HDAC6 level and cisplatin IC_50_ in 15 NSCLC cell lines. As shown in Figure 1I and S2, HDAC6 protein level positively correlated with cisplatin IC_50_. The spearman coefficient (0.64875) and *p*-value (0.0089) suggested that this correlation was statistically significant. We next asked whether depletion of HDAC6 in non-transformed cells could sensitize cells to cisplatin. As shown in Figure S3, cisplatin IC_50_ was significantly decreased in HDAC6 knockout embryonic fibroblasts (MEFs) compared with wild type MEFs. Overall, our results indicated that HDAC6 confers cisplatin resistance.

### Depletion of HDAC6 Augments Cisplatin-induced Apoptosis

**Figure 2:**
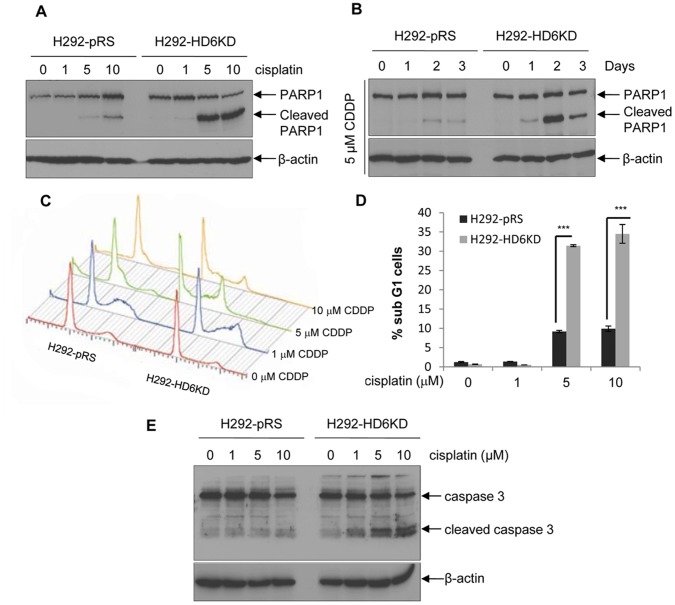
Depletion of HDAC6 augments cisplatin-induced apoptosis in H292 cells. A , H292-pRS or H292-HD6KD cells were treated with indicated concentrations of cisplatin for 24 hours. Anti-PARP1 and anti-β-actin Western blotting analyses were carried out. **B**, H292-pRS and H292-HD6KD cells were treated with 5 µM cisplatin for the indicated days. Anti-PARP1 and anti-β-actin Western blotting analyses were performed. **C**, Sub-G1 population was determined in H292-pRS or H292-HD6KD cells treated with vehicle (0 µM cisplatin) or cisplatin by FACS analysis. CDDP represents cisplatin. **D**, Quantitative presentation of the percentages of sub-G1 phase cells from **C**. **E**, H292-pRS or H292-HD6KD cells were treated with indicated dosage of cisplatin, and anti-cleaved caspase 3 and anti-β-actin Western blotting analyses were performed.

To determine whether knockdown of HDAC6 enhanced cisplatin cytotoxicity is due to cell apoptosis, we examined PARP1 cleavage in H292-pRS and H292-HD6KD pair. As shown in Figure 2A, cleaved PARP1 was elevated in H292-HD6KD cells compared with that in H292-pRS cells after 5 µM or 10 µM cisplatin treatment. In addition, as shown in Figure 2B, the cleaved PARP1 band appeared earlier (day 1) in H292-HD6KD cells compared with control cells (day 2). Similar results were also obtained using HDAC6 wild type and knockout MEFs pair (Figure S4) and A549-Sup and A549-HD6KD pair (data not shown). To confirm our results, flow cytometry analysis was employed to detect apoptotic population in cells exposed to cisplatin. As shown in Figure 2C and 2D, the sub-G1 population, which indicates apoptotic cells [35], was dramatically increased in H292-HD6KD cells compared with H292-pRS cells exposed to 5 or 10 µM cisplatin. Therefore, depletion of HDAC6 increased cisplatin-induced apoptosis. To examine whether the apoptosis was caspase 3 dependent, we detected the levels of cleaved caspase 3 in H292-pRS or H292-HD6KD cells exposed to cisplatin. As shown in Figure 2E, the levels of cleaved caspase 3 were increased in HD6KD cells when exposed to 1, 5 or 10 µM cisplatin, compared with control cells. Overall, our results suggest that depletion of HDAC6 increases caspase-dependent apoptosis upon cisplatin treatment in NSCLC cells.

### Knockdown of HDAC6 Exacerbates Cisplatin-induced DNA Damage

**Figure 3:**
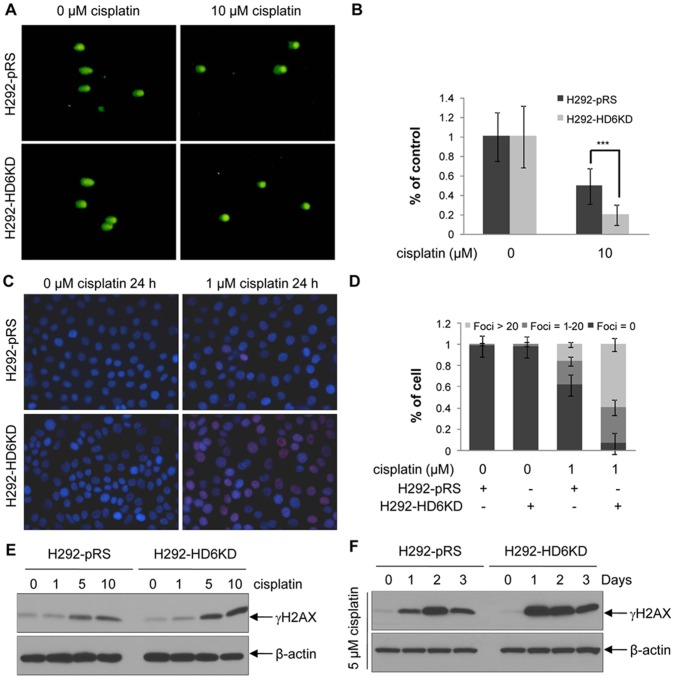
Knockdown of HDAC6 exacerbates cisplatin-induced crosslinking and accumulates more γH2AX foci in H292 cells. **A**, H292-pRS or H292-HD6KD cells were treated with vehicle (0 µM cisplatin) or cisplatin for 24 hours, then irradiated with 10 Gy of gamma radiation to introduce random single-strand breaks. Comet assays were performed as described in the Methods. **B**, Uncrosslinked DNA were scored by High Capacity Sides Analysis System (HCSA) software (Coats Associates, Inc.). Columns, mean from 40 single cells; bars, SE (***, P<0.001). **C**, Immunofluorescence staining of γH2AX foci in H292-pRS or H292-HD6KD cells treated with vehicle (0 µM cisplatin) or cisplatin as indicated. **D**, Quantitative representation of γH2AX foci in above cells. *Columns, mean from 600 cells.* **E**, H292-pRS and H292-HD6KD cells were treated with the indicated concentrations of cisplatin for 24 hours, and anti-γH2AX and anti-β-actin Western blotting analyses were then performed. **F**, The above cells were treated with vehicle or 5 µM cisplatin for the indicated days and anti-γH2AX and anti-β-actin Western blotting analyses were performed.

**Figure 4:**
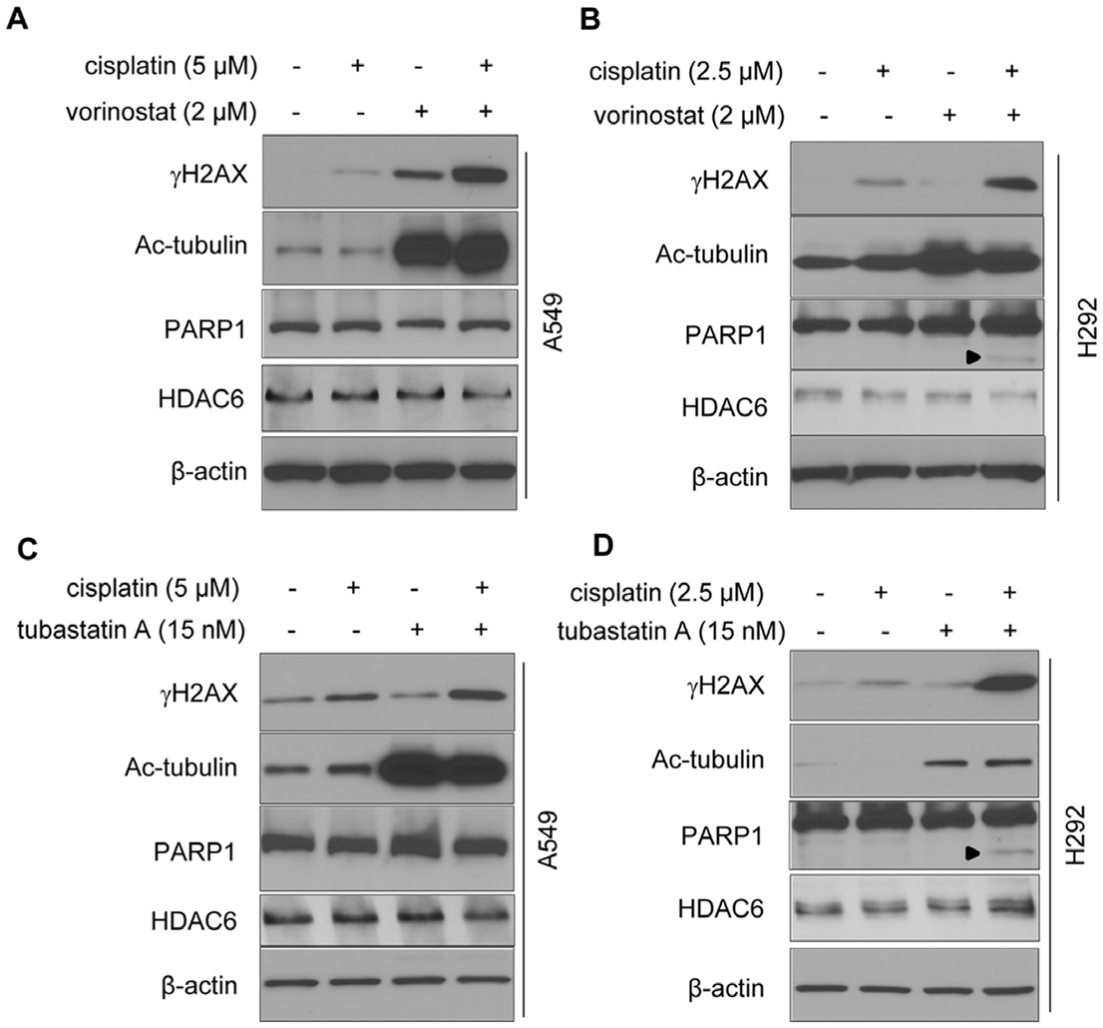
Inhibition of HDAC6 activity with pharmacological inhibitors enhances cisplatin-induced DNA damage in A549 and H292 cells. A , A549 cells were treated with vehicle control, cisplatin alone, vorinostat alone, or cisplatin and vorinostat in combination for 24 hours, then anti-γH2AX, anti-acetylated tubulin, anti-PARP1, anti-HDAC6, and anti-β-actin Western blotting analyses were performed. **B**, H292 cells were treated with vehicle control, cisplatin alone, vorinostat alone or cisplatin and vorinostat in combination for 24 hours, then anti-γH2AX, anti-acetylated tubulin, anti-PARP1, anti-HDAC6, and anti-β-actin Western blotting analyses were performed. The arrowhead indicated the cleaved PARP1 band. **C**, A549 cells were treated with vehicle control, cisplatin alone, tubastatin A alone or cisplatin and tubastatin A in combination for 24 hours, then anti-γH2AX, anti-acetylated tubulin, anti-PARP1, anti-HDAC6 and anti-β-actin Western blotting analyses were performed. **D**, H292 cells were treated with vehicle control, cisplatin alone, tubastatin A alone or cisplatin and tubastatin A in combination for 24 hours, and anti-γH2AX, anti-acetylated tubulin, anti-PARP1, anti-HDAC6 and anti-β-actin Western blotting analyses were performed. The arrowhead indicated the cleaved PARP1 band.

To explore the functions of HDAC6 in cisplatin-induced DNA damage, H292-pRS and H292-HD6KD cells were examined by single cell comet assay, a standard technique to evaluate DNA damage. Following cisplatin treatment, both cells were exposed to 10 Gy of ionizing radiation to induce random single strand breaks (indicated by comet tails). The efficacy of cisplatin-induced cross linking was shown by shortening of the comet tails [36]. As shown in Figure 3A, left two panels, after irradiation similar distinct comets were observed in both control and HD6KD cells exposed to vehicle control. However, upon cisplatin treatment, more shortened tails were observed in HD6KD cells compared with control cells (Figure 3A, right two panels), suggesting that depletion of HDAC6 sensitized H292 cells to cisplatin-induced DNA damage. Quantitative analysis of the comet assay was shown in Figure 3B. Cisplatin can also stimulate H2AX phosphorylation on serine 139 (γH2AX) whose foci formation can be used to analyze DNA damage [37]–[39]. We therefore used immunofluorescence staining to determine γH2AX foci formation. Following cisplatin treatment, more γH2AX foci were identified in H292-HD6KD cells compared with control cells (Figure 3C). The foci in the nucleus were categorized into three groups: Foci >20, Foci = 1∼20 and Foci = 0. The percentage of cells falling into the above three groups were quantified. As shown by Figure 3D, higher percentages of cisplatin-treated HD6KD cells belonged to the Foci >20 group, as compared with that of control cells, suggesting that depletion of HDAC6 stimulated cisplatin-induced DNA damage. Similarly, in A549-Sup and HD6KD or C13-pRS and HD6KD pairs, more foci>20 group were found in HDAC6 knockdown cells (data not shown). Consistently, Western blot analysis showed that the levels of γH2AX upon 5 or 10 µM cisplatin treatments were higher in H292-HD6KD cells compared with H292-pRS control cells (Figure 3E). In addition, the γH2AX levels were higher in H292 HDAC6 knockdown cells exposed to 5 µM cisplatin for 1, 2 and 3 days compared with control cells (Figure 3F). Similar results were also observed in A549 control and A549 HDAC6 knockdown pair (Figure S5). We next used pharmacological inhibitors such as vorinostat (pan-HDAC inhibitor) and tubastatin A (HDAC6-selective inhibitor) to validate that suppression of HDAC6 activity increases cisplatin-induced DNA damage in NSCLC cells. As shown in Figure 4A and 4B, co-treatment of vorinostat and cisplatin drastically increased the levels of γH2AX in A549 or H292 cells compared with the cells treated with either cisplatin or vorinostat alone. The similar results were also obtained in A549 and H292 cells using tubastatin A to replace vorinostat (Figure 4C and 4D). Both vorinostat and tubastain A can dramatically increase the levels of acetylated tubulin, suggesting that HDAC6 activity was effectively inhibited. Interestingly, apoptosis was detected as the cleaved PARP1 in H292 cells treated with cisplatin and vorinostat in combination or cisplatin and tubastatin A in combination but not in A549 cells (Figure 4). This data suggest that H292 cells are more sensitive to these drugs than A549 cells. HDAC6 protein levels were unaffected by drug treatment (Figure 4). Together, our results suggest that inhibition of HDAC6 activity sensitizes cisplatin-induced DNA damage.

### Knockdown of HDAC6 Increases Phosphorylation of DNA Damage Signaling Proteins Upon Cisplatin Treatment

**Figure 5:**
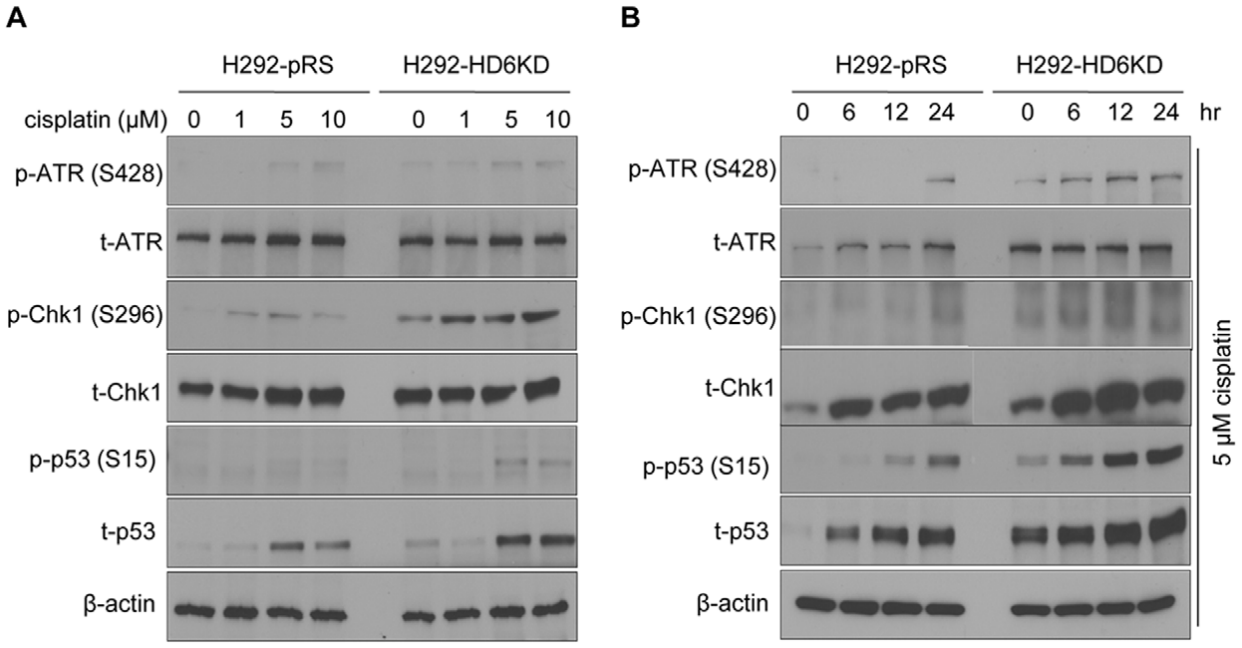
Knockdown of HDAC6 increases phosphorylation of DNA damage signaling proteins upon cisplatin treatment in H292 cells. **A**, H292-pRS and H292-HD6KD cells were treated with vehicle (0 µM cisplatin) or cisplatin for 24 hours, and anti-phosphorylated ATR (S428), total ATR, phosphorylated Chk1 (S296), total Chk1, phosphorylated p53 (S15), total p53 and β-actin Western blotting analyses were performed. **B**, The above cells were treated with 5 µM cisplatin for the indicated hours and Western blotting analyses with indicated antibodies were carried out.

The major molecular sensors of DNA damage include ATM (ataxia telangiectasia mutated) and ATR (ataxia telangiectasia and Rad3-related) [40], [41]. In response to DNA damage, these kinases will recruit and activate other signaling proteins, such as Chk1 and Chk2, inducing cell cycle arrest or apoptosis. All these kinases, ATM, ATR, Chk1 and Chk2 can phosphorylate and activate p53 which leads to cells undergoing apoptosis following DNA damage. To determine whether HDAC6 reduction could enhance DNA damage signaling after cisplatin treatment, the key proteins were tested. H292-pRS and H292-HD6KD cells were treated with vehicle (0 µM cisplatin) or cisplatin for 24 hours. As shown in Figure 5A, the levels of phospho-ATR (Ser428), phospho-Chk1 (Ser296), and phospho-p53 (Ser15) were increased in HDAC6 knockdown cells than in control cells (Figure 5A) suggesting that ATR/Chk1 signaling was more active upon cisplatin treatment in HDAC6 knockdown cells than that in control cells. Similar results were observed when cells were treated with cisplatin for 6, 12, and 24 hours (Figure 5B). In addition, we examined the phospho-ATM (Ser1981), phospho-Chk2 (Thr68) and phospho-p53 (Ser20) following cisplatin treatment, no significant change was found between H292 control and HDAC6 knockdown cells (data not shown), suggesting that depletion of HDAC6 did not augment ATM/Chk2 pathway. These results indicated that cisplatin is more effective in inducing DNA damage signaling in HDAC6 knockdown cell line than in the control cell line.

### Knockdown of HDAC6 Inhibits Tumor Growth in a Mouse Xenograft Model

**Figure 6:**
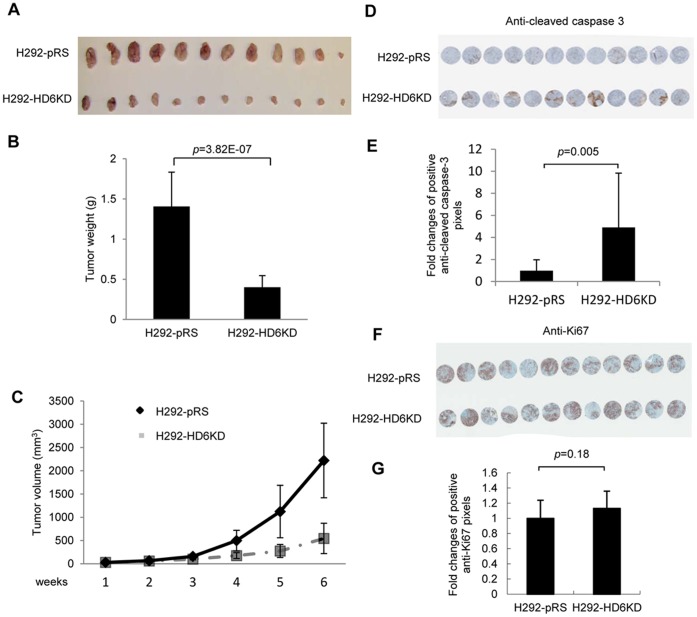
Knockdown of HDAC6 inhibits tumor growth in a mouse xenograft model. A and B , H292-pRS or H292-HD6KD cells were injected into 5- to 6-week-old athymic nude female mice (Nu/Nu mice) as described in the Methods. After 6 weeks, tumors were taken from the mice (**A**) and weighed (**B**). *Columns, mean from 12 mice/group.* **C**, The size of H292-pRS and H292-HD6KD xenografts were calculated weekly as described in the Methods. **D**, A tissue microarray of H292-pRS and H292-HD6KD xenografts was constructed and stained with anti-cleaved caspase 3 antibodies as described in the Methods. **E**, The intensity of cleaved caspase 3 staining was measured by pixels as described in the Methods. *Columns, mean from 12 xenografts/group.* **F**, A tissue microarray of H292-pRS and H292-HD6KD xenografts as shown in **D** was stained with anti-Ki67 antibodies as described in the Methods. **G**, The intensity of anti-Ki67 staining was measured by pixels as described in the Methods. *Columns, mean from 12 xenografts/group.*

To investigate the effect of HDAC6 knockdown on tumor growth *in vivo*, we injected H292-pRS or H292-HD6KD cells into nude mice and analyzed the tumor development by xenograft. The results showed that knockdown of HDAC6 significantly reduced tumor volume and weight (Figure 6A–6C), indicating that HDAC6 is essential to promote tumor growth in a xenograft mouse model. To further determine whether the reduced tumor growth of H292-HD6KD is due to decreased proliferation or increased apoptosis, we examined the cleaved caspase 3 and Ki67 levels in H292-pRS and H292-HD6KD xenografts. As shown in Figures 6D and 6E, increased cleaved caspase 3 levels were detected in H292-HD6KD xenografts compared with control xenografts. As shown in Figures 6F and 6G, there was no significant difference of Ki67 level between H292-pRS and H292-HD6KD xenografts. These results suggest that depletion of HDAC6 reduced tumor growth is due to increased basal pro-apoptotic signaling rather than decreased proliferation.

## Discussion

Here, we report that HDAC6 plays an important role in cisplatin resistance in NSCLC cell lines. Interestingly, we also found that HDAC6-depleted H292 cells formed smaller colonies in cell culture (Figure 1G) and smaller xenografts in nude mice compared to control cells (Figure 6). These findings suggest that effective inhibition or depletion of HDAC6 in NSCLC will not only benefit NSCLC patients undergoing platinum-based chemotherapy but may also simply reduce tumor growth in patients.

Because the majority of HDAC6 resides in the cytoplasm and most of HDAC6 substrates such as α-tubulin, hsp90 and cortactin are localized in the cytosol [14]–[16], the role of HDAC6 in DNA damage response has long been understudied. Until recently, HDAC6 involvement in response to DNA damaging anti-cancer agents began to emerge. It was shown that specific inhibition of HDAC6 by RNAi resulted in increased radio sensitivity in breast cancer cells [42]. In addition, a recent report showed that HDAC6-selective inhibitor tubacin significantly enhanced cell death induced by the topoisomerase II inhibitors etoposide and doxorubicin and the pan HDAC inhibitor SAHA (vorinostat) in transformed LNCap and MCF7 cells, but not in non-transformed cells [38]. However, we showed that the non-transformed HDAC6 knockout MEFs were more sensitive to cisplatin than their HDAC6 wild type counterparts by MTT assay and assessing the PARP1 cleavage (Figure S3 and S4). This discrepancy is probably due to the different anti-cancer agent and different non-transformed cell lines utilized in our studies. In addition, we found that depletion of HDAC6 stimulated the DNA damage response through the ATR/Chk1 rather than ATM/Chk2 pathway. We did not observe the phenomenon of Chk1 degradation via the ubiquitin proteasome pathway following cisplatin treatment as described by Namda et al [38]. Again, this could be due to the different cancer cells we used in this study.

It is intriguing why HDAC6 knockdown renders enhanced DNA damage response as is evidenced by increased ATR and ChK1 activity (Figure 5). Our preliminary data showed that a small portion of HDAC6 was translocated to the nucleus following the treatment of DNA- damaging drugs (Xiang et al., unpublished data). It is very tempting to hypothesize that HDAC6 may directly or indirectly affect ATR or ChK1 acetylation status which alters their phosphorylation and activity. This line of research is currently underway.

It is well documented that activation of the p53 pathway could sensitize cells to cisplatin, leading to cell cycle arrest and apoptosis [43], [44]. In agreement with this, the functionality of the p53-activated pathway has been shown to positively correlate with the cytotoxicity of all platinum compounds in NCI panel of 60 human tumor cell lines [45]. Consistent with the literature, we presented that H292 cells depleted of HDAC6 showed an increase of total p53 levels, as well as p53 phosphorylation on Serine 15 (Figure 5). Several kinases such as ATM, ATR, p38 and ERK1/2 are involved in phosphorylating p53 on serine 15 [46]–[49]. We showed that ATR was more active following cisplatin treatment in H292 HDAC6 knockdown cells than that in control cells, suggesting that HDAC6 depletion induces ATR activation and ATR may be responsible for p53 Serine 15 phosphorylation. Early investigation showed that in HDAC6 depleted A549 cells, ERK1/2 activities were increased upon EGF stimulation [50]. Therefore, ERK1/2 might also target p53 phosphorylation. Future experiments employing the kinase inhibitors and RNAi approach will elucidate which kinase is responsible for enhanced phosphorylation of p53 serine 15 in H292 HDAC6 knockdown cells. Since phosphorylation of p53 on serine 15 reduces its interaction with the negative regulator MDM2 [51], HDAC6-depletion mediated p53 phosphorylation prolongs its stability which may account for the increase of total p53 level in the HDAC6 knockdown cells. It is interesting to note that among the 15 NSCLC cell lines we have examined for HDAC6 levels and cisplatin IC_50_, 9 of them harbor p53 deletion or mutation. This observation suggests that inhibition or depletion of HDAC6 can sensitize p53 negative NSCLC following cisplatin treatment. Currently, the mechanisms of this observation are being investigated.

Although it is well accepted that HDACs play import roles in chemoresistance, there is very few literature that provide in-depth mechanisms of HDAC-conferred resistance. Recently, Edmond *et al.,* reported that acetylation and phosphorylation of a splicing factor, SRSF2, controlled cell fate decision in response to cisplatin [52]. HDAC6 was responsible for deacetylation and stabilization of SRSF2. SRSF2 together with its kinase SRPK2 controlled the splicing of caspase-8 to generate the pro-apoptotic form, leading to apoptosis in cells upon cisplatin treatment. These observations seem to contradict our conclusion that HDAC6 confers resistance. To examine whether HDAC6 level positively correlates to SRSF2 level, we performed anti-HDAC6 and anti-SRSF2 Western Blotting analysis in 11 of our 15 NSCLC cell lines. Our results showed no positive correlation between HDAC6 and SRSF2 in those cell lines (Data not shown). Since regulation of SRSF2 involved acetyltransferase Tip60, and kinases SRPK1 and SRPK2 [52], it is conceivable to speculate that in most tumor cells the regulation of SRSF2 was disturbed. In addition, this study did not perform the experiment to show that HDAC6 wild type cell lines were more sensitive to cisplatin compared with HDAC6KO or HDAC6KD cells. We assume that HDAC6 has several targets involved in cisplatin response. Therefore, the functionality of HDAC6 to stabilize SRSF2 and induce apoptosis following cisplatin treatment may not reflect the net effect of HDAC6 functionality in response to cisplatin. Further in-depth analysis on HDAC6 and SRSF2 is warranted.

Paclitaxel, which interferes with the normal breakdown of microtubules during cell division by stabilizing microtubules [53], is usually combined with cisplatin in clinical applications. Our results indicate that HDAC6 depletion does not potentiate paclitaxel activity (Figure 1C and 1F), suggesting that HDAC6 depletion may collaborate with cisplatin using a different mechanism other than stabilizing microtubules. It was reported that combining vorinostat with paclitaxel could enhance paclitaxel functions [54], which may be due to the inhibition of other HDACs by vorinostat. Apart from paclitaxel, we also examined the correlation between HDAC6 protein level and IC_50_ of other drugs such as gemcitabine and pemetrexed from our previous work [55]. No significant correlation was found, suggesting that HDAC6 may be involved in drug resistance specific for cisplatin.

Although HDAC6 overexpression was found in certain types of cancer, the role of HDAC6 in tumorigenesis has not been established until the report by Dr. T-P Yao’s group [56]. In their study, tumor growth of HDAC6 knockdown ovarian cancer cells was significantly retarded as compared with that of control cancer cells. Consistent with their investigation, we showed similar results in an HDAC6 knockdown NSCLC cell line (Figure 6), suggesting that this observation is not tumor type specific. Since tumor growth was significantly reduced in H292 HDAC6 knockdown xenografts, it will be difficult to study cisplatin sensitivity *in vivo* using this cell line. Future study by using an inducible H292 HDAC6 knockdown cell line may overcome this problem. Overall, our studies suggest that HDAC6 confers cisplatin resistance in NSCLC cells. HDAC6 could be a novel therapeutic target in NSCLC intervention.

## Supporting Information

Figure S1 **Depletion of HDAC6 in C13 cells re-sensitizes C13 cells to cisplatin. A,** C13 cells were stably transfected with control shRNA vector or HDAC6 shRNA vector (Origene) to generate C13-pRS or C13-HD6KD clone, respectively. Anti-HDAC6 and anti-β-actin Western blotting analyses were performed as indicated. **B,** A three-day MTT assay using C13-pRS and C13-HD6KD cells was performed. ***, denoted *p*<0.001. Cisplatin IC_50_ in those cells was also shown.
(TIF)

Figure S2 **HDAC6 expression in a panel of NSCLC cells.** Anti-HDAC6 and anti-β-actin Western blotting analyses were performed using a panel of NSCLC cells. HDAC6 expression was quantified by densitometry analysis. The cisplatin IC_50_ was calculated by Origin 75 software.
(TIF)

Figure S3 **HDAC6 knockout MEFs are more sensitive to cisplatin than their HDAC6 wild type counterparts. A**, HDAC6 protein level was detected in HDAC6 wild type (WT) and knockout (KO) MEFs by Western blotting analysis using anti-mouse HDAC6 antibodies (a kind gift from Dr. Tso-Pang Yao). **B**, A three-day MTT assay using HDAC6 WT and HDAC6 KO MEFs was performed. ***, denoted *p*<0.001. The cisplatin IC_50_ was calculated by Origin 75 software.
(TIF)

Figure S4 **HDAC6 knockout MEFs display enhanced apoptosis upon cisplatin treatment compared with their wild type counterparts.** HDAC6 WT and KO MEFs were treated with 10 µM cisplatin at the indicated time intervals and apoptotic phenotypes were examined by anti-PARP1 Western blotting analysis.
(TIF)

Figure S5 **Knockdown of HDAC6 exacerbates cisplatin-induced DNA damage in A549 cells.** **A,** Immunofluorescence staining of γH2AX in A549-Sup and A549-HD6KD cells treated with vehicle (0 µM cisplatin) or cisplatin as indicated. **B,** A549-Sup and A549-HD6KD cells were treated with the indicated concentrations of cisplatin for 24 hours (upper panels) or with 10 µM cisplatin for the indicated days (lower panels). Anti-γH2AX and anti-β-actin Western blotting analyses were then performed.
(TIF)
