# Supplementary figures and images for: Depletion of HDAC6 Enhances Cisplatin-Induced DNA Damage and Apoptosis in Non-Small Cell Lung Cancer Cells
Source: PLoS One. 2012 Sep 5;7(9):e44265. doi: 10.1371/journal.pone.0044265 (PMC3434198; doi:10.1371/journal.pone.0044265)

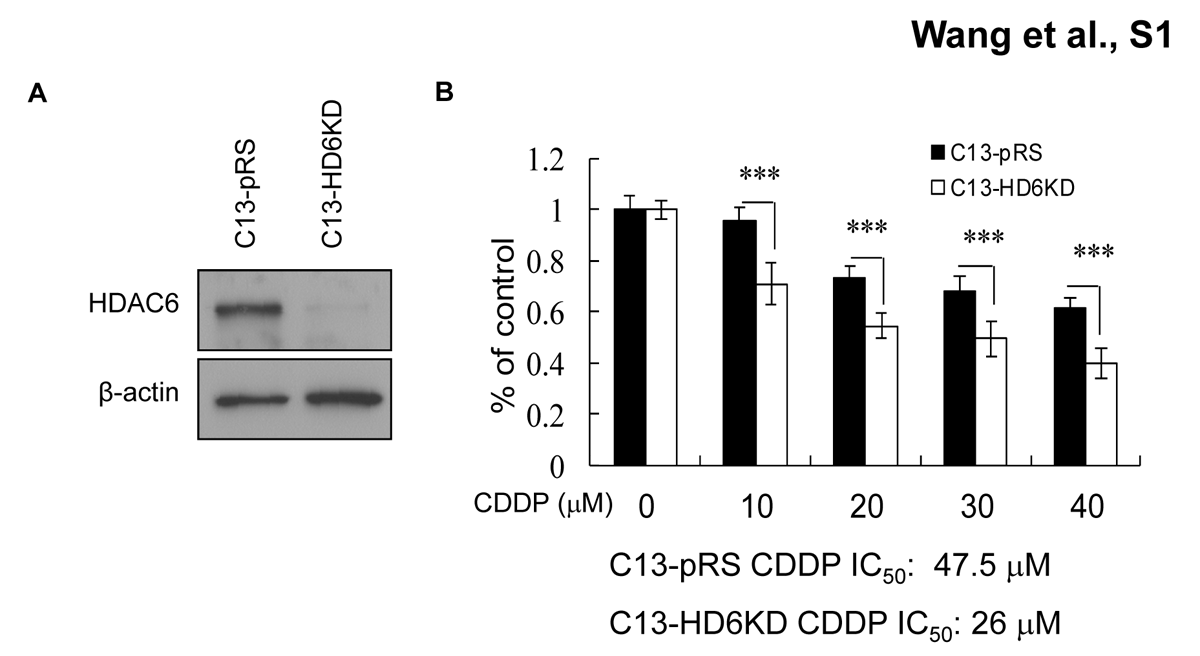

Supplement: Figure S1 — Depletion of HDAC6 in C13 cells re-sensitizes C13 cells to cisplatin. A, C13 cells were stably transfected with control shRNA vector or HDAC6 shRNA vector (Origene) to generate C13-pRS or C13-HD6KD clone, respectively. Anti-HDAC6 and anti-β-actin Western blotting analyses were performed as indicated. B, A three-day MTT assay using C13-pRS and C13-HD6KD cells was performed. ***, denoted p<0.001. Cisplatin IC50 in those cells was also shown. (TIF) [file pone.0044265.s001.tif]

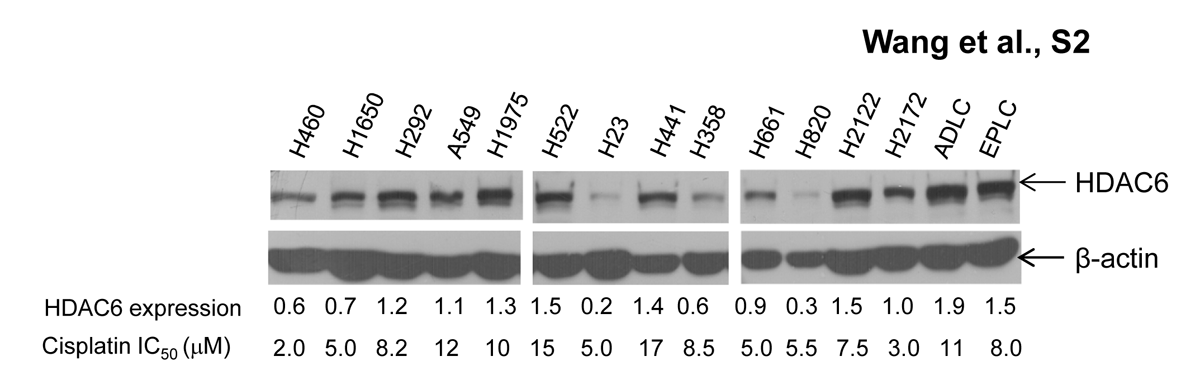

Supplement: Figure S2 — HDAC6 expression in a panel of NSCLC cells. Anti-HDAC6 and anti-β-actin Western blotting analyses were performed using a panel of NSCLC cells. HDAC6 expression was quantified by densitometry analysis. The cisplatin IC50 was calculated by Origin 75 software. (TIF) [file pone.0044265.s002.tif]

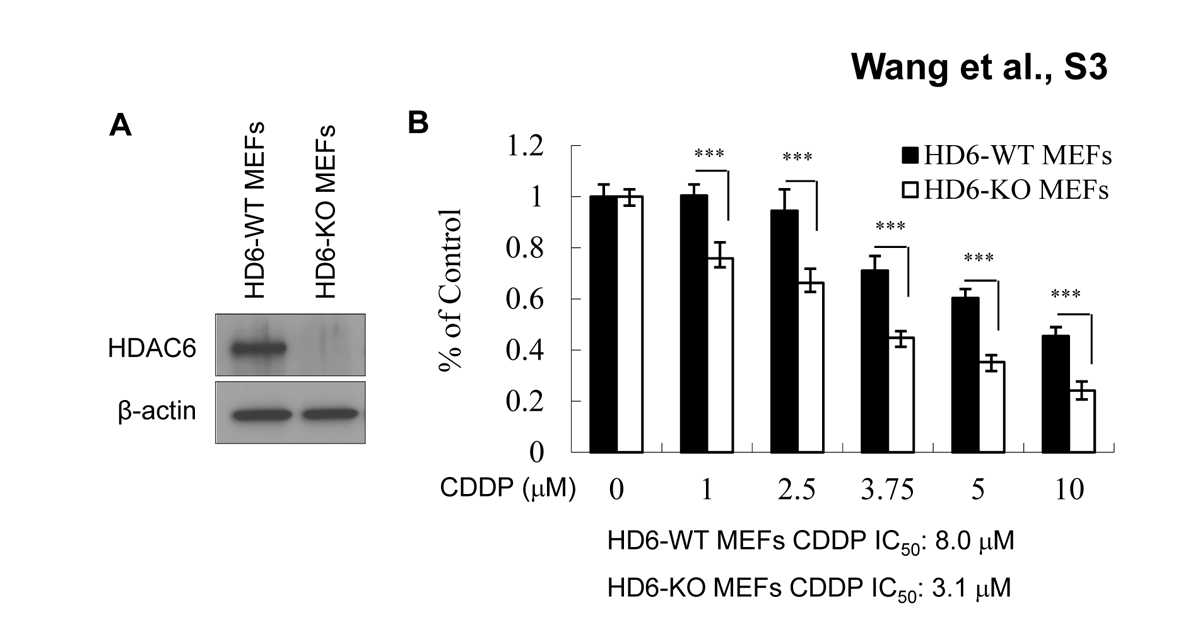

Supplement: Figure S3 — HDAC6 knockout MEFs are more sensitive to cisplatin than their HDAC6 wild type counterparts. A, HDAC6 protein level was detected in HDAC6 wild type (WT) and knockout (KO) MEFs by Western blotting analysis using anti-mouse HDAC6 antibodies (a kind gift from Dr. Tso-Pang Yao). B, A three-day MTT assay using HDAC6 WT and HDAC6 KO MEFs was performed. ***, denoted p<0.001. The cisplatin IC50 was calculated by Origin 75 software. (TIF) [file pone.0044265.s003.tif]

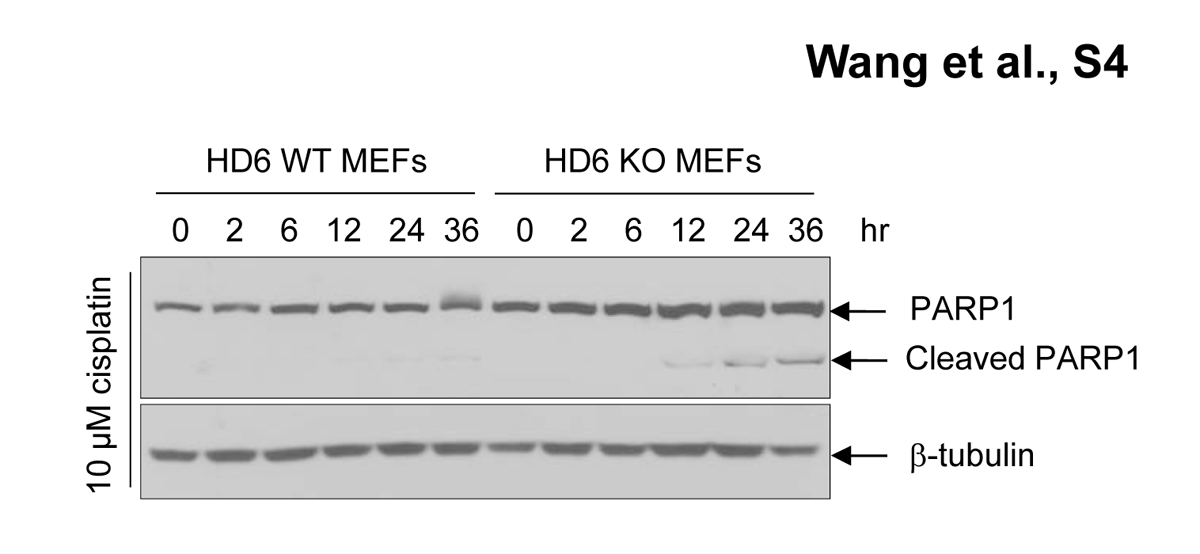

Supplement: Figure S4 — HDAC6 knockout MEFs display enhanced apoptosis upon cisplatin treatment compared with their wild type counterparts. HDAC6 WT and KO MEFs were treated with 10 µM cisplatin at the indicated time intervals and apoptotic phenotypes were examined by anti-PARP1 Western blotting analysis. (TIF) [file pone.0044265.s004.tif]

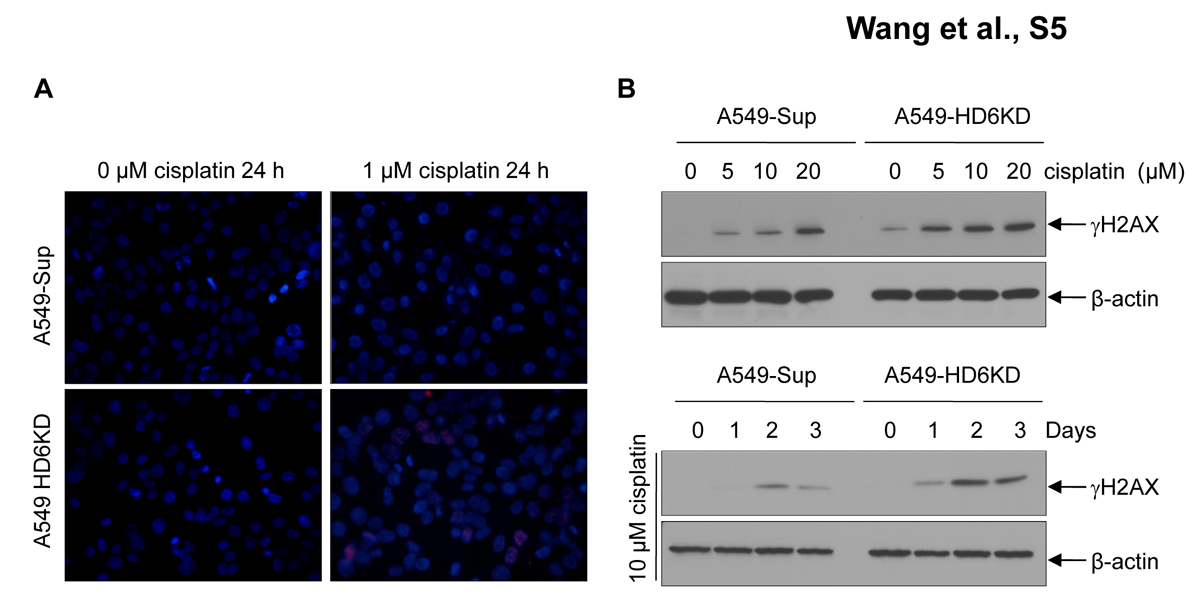

Supplement: Figure S5 — Knockdown of HDAC6 exacerbates cisplatin-induced DNA damage in A549 cells. A, Immunofluorescence staining of γH2AX in A549-Sup and A549-HD6KD cells treated with vehicle (0 µM cisplatin) or cisplatin as indicated. B, A549-Sup and A549-HD6KD cells were treated with the indicated concentrations of cisplatin for 24 hours (upper panels) or with 10 µM cisplatin for the indicated days (lower panels). Anti-γH2AX and anti-β-actin Western blotting analyses were then performed. (TIF) [file pone.0044265.s005.tif]
